# Cardiac hypertrophy or failure? - A systematic evaluation of the transverse aortic constriction model in C57BL/6NTac and C57BL/6J substrains

**DOI:** 10.1016/j.crphys.2019.10.001

**Published:** 2019-11-02

**Authors:** Min Zi, Nicholas Stafford, Sukhpal Prehar, Florence Baudoin, Delvac Oceandy, Xin Wang, Thuy Bui, Mohamed Shaheen, Ludwig Neyses, Elizabeth J. Cartwright

**Affiliations:** Division of Cardiovascular Sciences, University of Manchester and Manchester Academic Health Science Centre, Manchester, United Kingdom

**Keywords:** C57BL/6, Cardiac hypertrophy, Heart failure, Transverse aortic constriction (TAC)

## Abstract

**Background:**

The mouse model of transverse aortic constriction (TAC) has been widely used as a cardiac stress in the investigation of the molecular mechanisms of cardiac hypertrophy. Recently, the International Knockout Mouse Consortium has selected the C57BL/6NTac (BL/6N) mouse strain to generate null alleles for all mouse genes; however, a range of genetic and cardiac phenotypic differences have been reported between this substrain and the commonly used C57BL/6J (BL/6J) substrain. It has been reported by Garcia-Menendez and colleagues that 12-week C57BL/6NTac mice are susceptible to heart failure but little is known about the cardiac remodeling in this substrain as cardiac function progresses from compensation to decompensation.

**Methods:**

BL/6J and BL/6N mice were subjected to pressure overload via TAC. The impact of both age and duration of cardiac pressure overload induced by TAC on cardiac remodelling were systematically assessed.

**Results:**

Our data showed that BL/6N mice developed eccentric hypertrophy with age- and time-dependent deterioration in cardiac function, accompanied by considerable interstitial fibrosis. In contrast, BL/6J mice were more resilient to TAC-induced cardiac stress and developed variable cardiac phenotypes independent of age and the duration of pressure overload. This was likely due to the greater variability in pre-TAC aortic arch dimension as measured by echocardiography. In addition to increased expression of brain natriuretic peptide and collagen gene type 1 and 3, BL/6N mice also had greater angiotensin II type 2 receptor (AT2R) gene expression than BL/6J counterparts at baseline and after 2-weeks TAC, which may contribute to the exacerbated interstitial fibrosis.

**Conclusions:**

BL/6N and BL/6J mice have very different responses to TAC stimulation and these differences should be taken into consideration when using the substrains to investigate the mechanisms of hypertrophy and heart failure.

## Highlights

• The first study to show that adult BL/6NTac mice have an age-dependent cardiac response to pressure overload.
• The first study to show BL/6NTac mice have a time-dependent cardiac response to pressure overload.
• C57BL/6J mice have variable cardiac remodelling that positively correlates with the original size of the aorta.

## Graphical abstract

**Figure:**
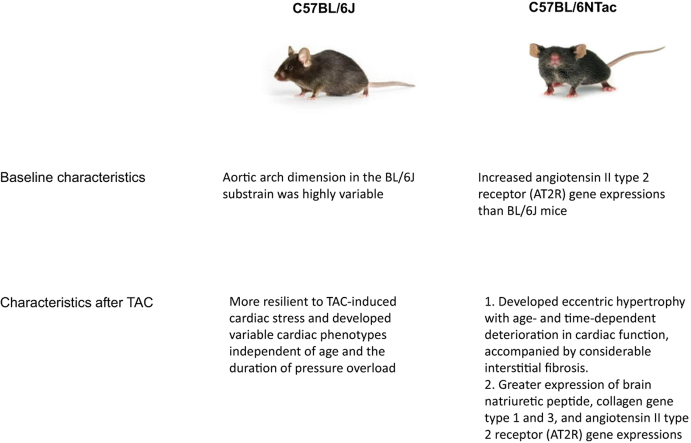

## 1 Introduction

Left ventricular (LV) hypertrophy is a critical consequence of systemic hypertension, aortic stenosis, hypertrophic cardiomyopathy, and itself is an independent risk factor for coronary heart disease, malignant arrhythmia, sudden death, and heart failure (Casale et al., 1986; Koren et al., 1991; Levy et al., 1989; Levy et al., 1990; Liao et al., 1995; Kahan and Bergfeldt, 2005). Therefore, targeting cardiac hypertrophy is essential for preventing adverse cardiovascular events. Several animal models of cardiac pathological hypertrophy, including isoproterenol or angiotensin II infusion, and aortic constriction, have been developed to investigate the mechanisms underlying hypertrophy and to characterise new therapies. In the mouse transverse aortic constriction (TAC) is used to induce pressure overload and somewhat mimic the condition of hypertension in humans, causing LV hypertrophy and subsequent heart failure (Rockman et al., 1991). This model is considered to be an excellent experimental tool and has been widely used in cardiovascular research over the past two decades, resulting in considerable advances in our understanding of the mechanisms underlying cardiac hypertrophy. However, the TAC results are often inconsistent from one laboratory to another despite the same degree of aortic constriction apparently being applied. Some investigators reported that C57BL/6 mice developed concentric cardiac hypertrophy with preserved cardiac function after 8 weeks of TAC (Garcia-Menendez et al., 2013) whilst others showed that C57BL/6 mice developed eccentric cardiac hypertrophy with deterioration of cardiac function 2–8 weeks after TAC (Lai et al., 2012; Asakura et al., 2002; Kemi et al., 2008; Li et al., 2003; Barrick et al., 2007). Mohammed and co-workers found that C57BL/6 mice presented variable phenotypes in response to TAC, independent of the duration of TAC (up to 9 weeks) (Mohammed et al., 2012). These characteristics may indirectly influence the experimental results of phenotypes being studied. Because mice of different ages (from 7 weeks to 14 weeks) were used and the duration of TAC varied in these studies, it is difficult to determine which confounding factors in experimental procedure led to such variable outcomes. Therefore, a systematic assessment is needed.

C57BL/6 is one of the most important mouse strains for biomedical research. It has been used directly to investigate the impact of pharmacological intervention on cardiac remodelling. In the past few decades, a large number of genetically engineered mouse lines bred on the C57BL/6J background have become been widely used in research (Waterston et al., 2002). Recently, the International Knockout Mouse Consortium (IKMC) used a C57BL/6NTac mouse embryonic stem cell (ESC) line to create a modified ESC bank (Skarnes et al., 2011; Pettitt et al., 2009). These ESC lines are being used by the International Mouse Phenotyping Consortium (IMPC) to generate and carry out phenotypic screening of 5000 mouse mutant lines, the first step to achieving a comprehensive decoding of mammalian gene function (Brown and Moore, 2012). It is therefore anticipated that an increasing number of investigations to elucidate gene function in the heart will be carried out using mouse models on the C57BL/6NTac genetic background.

The fact that there may be differences in the closely related C57BL/6 substrains has not been considered by most investigators; however recent studies have identified important baseline phenotypic and genomic differences between C57BL/6NTac and C57BL/6J mice (Simon et al., 2013; Mekada et al., 2009). C57BL/6NTac mice seem more susceptible to cardiac dysfunction and death than C57BL/6J mice after 8 weeks TAC (Garcia-Menendez et al., 2013); however, there has yet to be a study conducted to fully assess the TAC model in C57BL/6NTac versus the C57BL/6J substrain. To understand whether experimental age has an impact on the hypertrophic response, we performed the TAC procedure on both C57BL/6NTac and C57BL/6J mice aged 8, 10 and 12 weeks and assessed cardiac function after 2 weeks. Thereafter we subjected 8 week old mice to TAC and analysed cardiac function, morphological changes and gene expression profiles 2 weeks and 5 weeks post-TAC to investigate the effect of time course of pressure overload on cardiac remodelling. Data obtained demonstrate that following 2 weeks of TAC the BL/6N substrain aged 10 or 12 weeks manifested cardiac dysfunction, whilst most mice aged 8 weeks had cardiac hypertrophy with preserved cardiac function concomitant with abundant fibrosis and increased gene expression of brain natriuretic peptide (BNP), collagen gene 1α1 (Col1α1) and collagen gene 3α1(Col3α1). After 5 weeks of TAC, most BL/6N mice developed heart failure. In contrast, BL/6J mice were more resilient to TAC-induced pressure overload and developed variable cardiac phenotypes in each age group and at each given time point. Furthermore, we detected higher expression of type 2 angiotensin II receptor (AT2R) in the BL/6N substrain compared to the BL/6J substrain under basal conditions and after 2 weeks of exposure to TAC. Together, our data demonstrates that BL/6N and BL/6J substrains respond differently to TAC when age and duration of stress are considered; providing for the first time a practical guide for future research using these mouse models of pressure overload.

## 2 Materials and methods

### 2.1 Animals and experimental protocol

Clinical and animal studies have indicated that cardiac remodelling in response to pressure overload is more favourable in females than in males, i.e., less fibrosis, left ventricular dilation and dysfunction (Montalvo et al., 2012; Villar et al., 2009; Fliegner et al., 2010; Kararigas et al., 2014; Skavdahl et al., 2005). Due to such gender differences in cardiac remodelling, most researchers only use male mice to study the mechanisms underlying pressure overload induced cardiac hypertrophy and failure, as we do in the present study. Male C57BL/6J mice (Charles River, UK) (weight: 8 weeks old, 24–30 g; 10 weeks old, 24–28 g; 12 weeks old, 26–30 g) and C57 BL/6NTac mice (Taconic Farm) (weight: 8 weeks old, 23–29 g; 10 weeks old, 23–30 g; 12 weeks old, 23–30 g) were kept in a pathogen-free facility at the University of Manchester. All mice were maintained in ventilated cages with free access to standard rodent chow and sterile water in a temperature-controlled environment (21 ± 2 °C, lights on from 7:00 a.m. to 7:00 p.m.). The animal studies were performed in accordance with the U.K. Animals (Scientific Procedures) Act, 1986 and institution guidelines for laboratory animal research.

All mice underwent echocardiography and ECG assessments and were then randomly assigned to TAC or sham groups. To assess the age effect on cardiac outcomes, 8, 10, and 12 week old mice were subjected to a 2-week TAC or sham operation. Echocardiography was performed 2 weeks after TAC to determine cardiac structure and function. To evaluate the effect of duration of TAC on cardiac outcomes, 8 week old mice were divided into two groups. In group one, *in vivo* haemodynamic analysis, ECG and echocardiography were performed 2 weeks after TAC (n = 5–14 per group). Mice were then euthanised to obtain heart weight and tibia length ratio. Heart tissues were kept for histology and molecular studies. In group two, mice were followed by ECG and echocardiography for up to 5 weeks (n = 5–8 per group) and then euthanised to obtain heart weight and tibia length ratio.

### 2.2 Transverse aortic constriction

Mice were anaesthetised with 3% isoflurane through an endotracheal intubation and the surgical field disinfected. Partial thoracotomy through the left second rib was performed under a surgical microscope. The transverse aorta was isolated from surrounding fat tissue with two fine tip 45° angled forceps. Using 7-0 prolene sutures, two double overhand knots were then tied around the aorta and an overlying 27-gauge needle between the innominate and left common carotid arteries. The needle was then removed in order to yield a constriction of 0.41 mm in diameter. The chest was then closed using 6.0 prolene sutures. For the sham procedure, the aortic arch was isolated and twined with a 7-0 prolene suture without ligation. After TAC, mice were injected with buprenorphine (0.1 mg/kg) intraperitoneally and allowed to recover on a heating pad. All TAC procedures were performed by the same experienced operator who was blinded to animal details. Total mortality rate due to surgery or an acute response to the ligation was less than 10%.

### 2.3 Echocardiography

Transthoracic echocardiography (TTE) was performed at baseline, 1 ​day, 2 weeks and 5 weeks post-TAC using an Acuson Sequoia C256 system (Siemens) and a 14-MHz probe. Mice were lightly anaesthetised with 1% isoflurane, maintaining the heart rate at approximately 450 beats per minute. The M-mode parasternal short-axis views were taken to determine left ventricular end-diastolic dimension (LVEDD) and end-systolic dimension (LVESD), posterior wall thickness in diastole (LVPWD) and systole (LVPWS), and interventricular septum thickness in diastole (IVSD) and systole (IVSS) over three cardiac cycles. The analysis was performed blinded to animal details. LV fractional shortening (FS) was calculated using the formula FS ​= ​[(LVEDD - LVESD)/(LVEDD)] x 100. Relative wall thickness (RWT) was calculated using the equation RWT = (IVSD ​+ ​LVPWD)/LVEDD. In addition, the aortic arch diastolic dimension was measured using M-mode tracing from the suprasternal view between the horizontal and descending segments of the arch (Supplementary Figure 1). Aortic constriction = (pre-TAC aortic arch dimension - aortic arch dimension 24 h after TAC)/pre-TAC aortic arch dimension x 100%. Based on a clinical study by Ganau et al. (Ganau et al., 1992), we defined concentric LV hypertrophy as increased heart weight/body weight (HW/BW) ratio, normal FS, increased RWT, and normal or reduced LVEDD. Eccentric LV hypertrophy was defined as increased heart HW/BW ratio, normal FS, decreased RWT, and increased LVEDD. Heart failure with reduced systolic function was defined as in a previous report by signs such as dyspnoea and body weight loss (approximately 10% decrease), reduced FS, and increased wet lung weight to body weight (LW/BW) ratio (Liu et al., 2011).

### 2.4 Electrocardiography (ECG)

To monitor cardiac rhythms at baseline and in hypertrophic conditions, heart rate was recorded by *in vivo* surface ECG in conscious mice using the ECGenie™ system (Mouse Specifics, USA) prior to and 2 weeks after TAC.

### 2.5 *In vivo* haemodynamic analysis

Under 1% isoflurane, *in vivo* haemodynamic analyses were performed using a protocol described previously with a 1.4 F pressure-volume catheter (SPR-839, Miller Instruments) and a pressure-volume system (Millar Instruments) (Oceandy et al., 2007).

### 2.6 Morphological and histological analysis

Hearts were cross-dissected into two parts for molecular and histological examination. Tissue for histology was fixed in 4% paraformaldehyde for at least 48 h, embedded in paraffin and sectioned at a thickness of 5 μm. Haematoxylin & eosin and Masson's trichrome staining were performed using standard procedures. Image J software (National Institutes of Health, USA) was used to calculate the mean cross-sectional area by measuring 200 randomly selected cardiomyocytes from 5 sections per heart and to quantify the degree of myocardial interstitial fibrosis by measuring the percentage of Masson's Trichrome stained area from 60 randomly chosen frames from 5 sections per heart.

### 2.7 Immunohistochemistry

To assess capillary density immunohistochemistry was performed on paraffin embedded heart sections. Following the standard protocol, the endothelial cells were stained with anti-mouse CD31 monoclonal primary antibody (Pharmingen), followed by a biotinylated anti-mouse IgG secondary antibody, and an avidin-HRP conjugate for the colour reaction (DAB paraffin IHC staining module, Ventana Medical Systems, Inc). Capillaries were defined as brown round structures with a central lumen and a diameter less than 10 μm under light microscopy. Capillary density was defined as capillary to cardiomyocyte ratio.

### 2.8 Quantitative real-time polymerase chain reaction (qPCR)

Total RNA was extracted from the heart tissue using TRIzol (Invitrogen). After DNAse treatment, 500 ng of total RNA was reverse transcribed using the High-Capacity cDNA Archive Kit (Applied Biosystems). The expression of BNP, Col1α1 and Col3α1, β1 and β2 adrenoceptors (β1-AR and β2-AR), angiotensin II receptor type 1 (AT1R), and angiotensin II receptor type 2 (AT2R) was determined by real-time quantitative PCR using a 7500 Fast Real-Time PCR detection system (Applied Biosystems), and the results were analysed using the 2-ΔΔ^CT^ method (Livak and Schmittgen, 2001). All primers were obtained from Qiagen. GAPDH and β-actin were used as internal controls.

### 2.9 Statistical analysis

Data are expressed as mean ± S.E.M. and analysed using SPSS 20.0 software. Two-tail Student's t-test or Chi square test was used for the comparisons between the two groups. One-way ANOVA followed by Bonferroni corrected post-hoc *t*-test was used for the comparisons among multiple groups. Bivariate correlation analysis was conducted to determine the impact of age and time course on cardiac function as well as the correlation between HW/TL ratio and pre-TAC aortic arch dimension. F test was performed to compare the variability of two groups of data. Univariate analysis of variance has been performed to make sure the n number of mice is appropriate to show the significance. P-value <0.05 was considered statistically significant.

## 3 Results

### 3.1 The impact of age on strain-specific difference in cardiac function after TAC

**Fig. 1:**
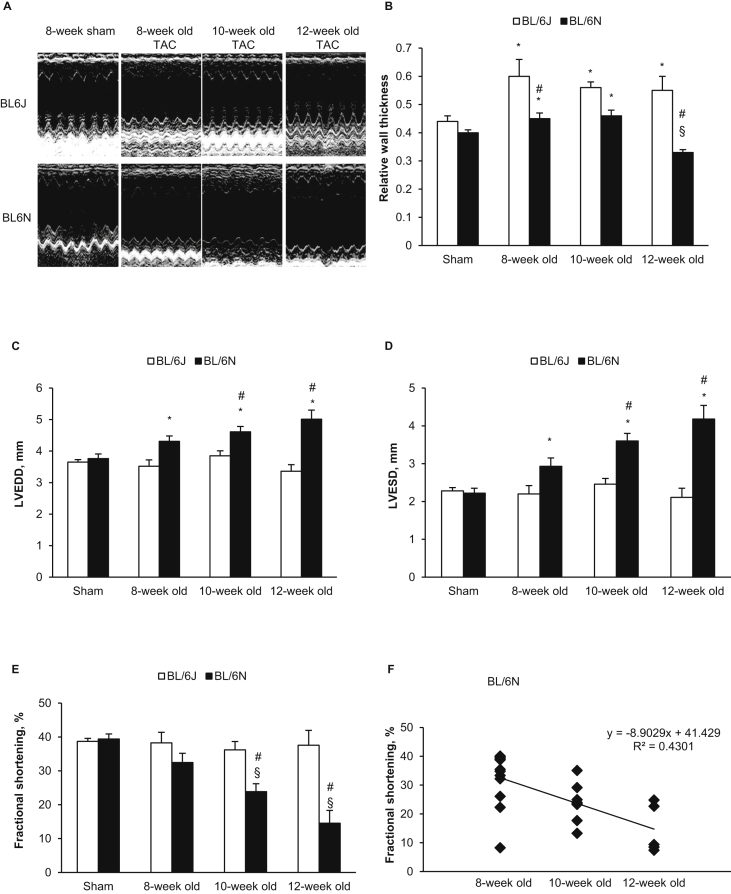
**Echocardiography analysis in different age groups after 2-week TAC and sham surgery. (A)** Example echocardiography of left ventricle. **(B)** Left ventricular relative wall thickness (RWT). **(C)** Left ventricular end-diastolic internal dimension (LVEDD). **(D)** Left ventricular end-systolic internal dimension (LVESD). **(E)** Left ventricular fractional shortening (FS). **(F)** In C57BL/6N TAC mice, FS decreased in an age dependent manner (R^2^ ​= ​0.430, p ​= ​0.003). Data are shown as means ​± ​SE (n ​= ​8 for sham and n ​= ​6–14 per group for TAC). *P ​< ​0.05 compared with the sham group of same strain, showing significant increase; §P ​< ​0.05 compared with the sham group of same strain, showing significant decrease; #P ​< ​0.05 compared with C57BL/6J mice of the same age group.

In previous studies, BL/6 mice aged 6–16 weeks have been used to investigate the effect of pressure overload on cardiac remodelling. We therefore subjected BL/6N and BL/6J mice aged 8, 10 and 12 weeks–2 weeks TAC, followed by echocardiographic assessment of cardiac structure and function (Fig. 1A). In BL/6N mice, RWT was significantly increased in the 8-week and 10-week groups compared with sham controls but reduced in the 12-week group compared with sham and BL/6J counterparts (Fig. 1B). LV was dilated as indicated by a significant increase in LVEDD (Fig. 1C) and LVESD (Fig. 1D) compared with sham controls (aged 8 weeks). Systolic function as assessed by FS was preserved in the 8-week group but was significantly reduced in the 10 and 12-week groups in an age-dependent manner (R^2^ = 0.430, p = 0.003) (Fig. 1E, F). Of note, the reduction was more prominent in 12-week mice with 5/5 mice exhibiting signs of heart failure (p = 0.018 comparing the three age groups) (Table 1), suggesting that the reduction in RWT in this age group was likely due to eccentric hypertrophy and dilation associated with heart failure. In contrast, BL/6J mice developed concentric hypertrophy indicated by greatly increased RWT (Fig. 1B) and unchanged LV internal dimension (Fig. 1C, D) and cardiac function (Fig. 1E) in all age groups subjected to TAC. These results suggest that age is a critical factor contributing to TAC-induced cardiac dysfunction in BL/6N mice but not in BL/6J mice.

**Table 1:** Heart failure rate in different age groups of mice after 2-week TAC.

Age (week)	C57BL/6J	C57BL/6N	P value
8	20% (2/10)	25% (3/12)	1.000
10	17% (1/6)	50% (3/6)	0.546
12	20% (1/5)	100% (5/5)	0.048
P value	1.000	0.018	

Heart failure with reduced systolic function was defined as having dyspnea, body weight loss (approximately 10% decrease), increased wet lung weight and reduced fractional shortening on echocardiography compared with sham controls.

### 3.2 The impact of the duration of TAC on strain-specific difference in cardiac function

**Fig. 2:**
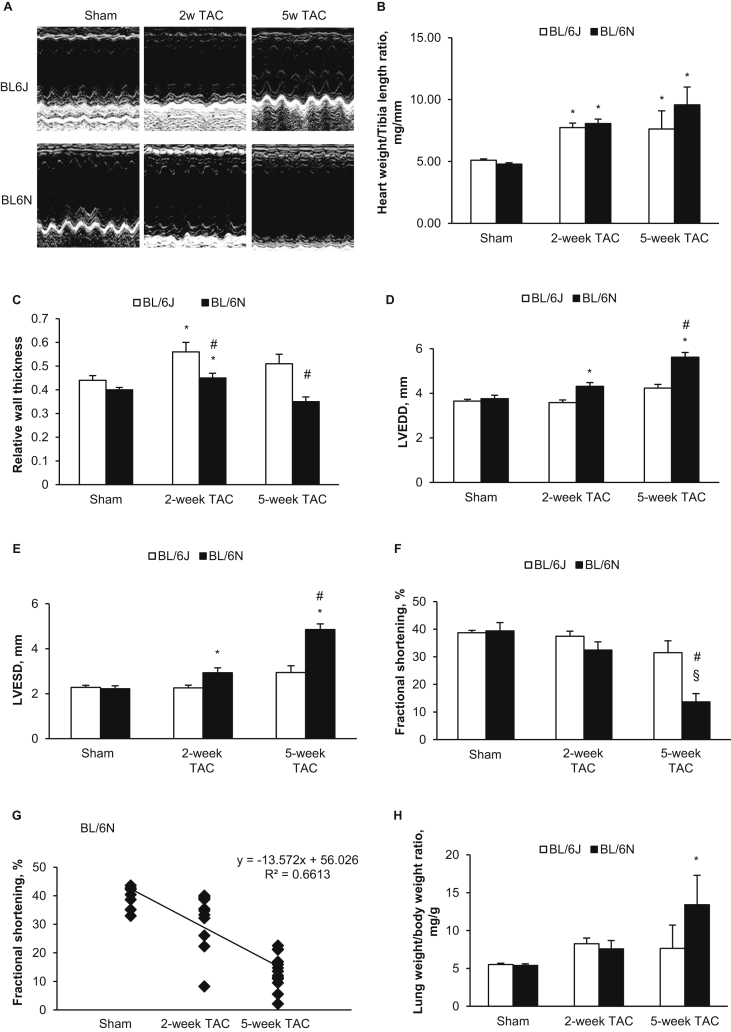
**Morphology and echocardiography analysis in 8 weeks old mice after TAC and sham surgery. (A)** Example echocardiography of left ventricle. **(B)** Heart weight to tibia length ratio. **(C)** Left ventricular relative wall thickness (RWT). **(D)** Left ventricular end-diastolic internal dimension (LVEDD). **(E)** Left ventricular end-systolic internal dimension (LVESD). **(F)** Left ventricular fractional shortening (FS). **(G)** In C57BL/6N TAC mice, FS decreased in time-dependent manner (R^2^ = 0.661, p < 0.0001). **(H)** Lung weight to body weight ratio. Data are shown as means ​± ​SE (n ​= ​8 for sham and n ​= ​5–14 per group for TAC). *P ​< ​0.05 compared with the sham group of same strain, showing significant increase; §P ​< ​0.05 compared with the sham group of same strain, showing significant decrease; #P ​< ​0.05 compared with C57BL/6J mice at the same time point of TAC.

TAC for a duration of eight weeks has been reported to induce heart failure in C57BL/6NTac mice (Garcia-Menendez et al., 2013). Therefore, to evaluate the temporal hypertrophic response, we challenged 8 week old mice to 2 and 5 weeks TAC (n = 5–12 per group, Fig. 2A). BL/6N mice developed eccentric hypertrophy after 2 weeks, indicated by significantly increased heart weight to tibia length (HW/TL) ratio (Fig. 2B), increased RWT (Fig. 2C), and dilated LV (Fig. 2D, E). Mean FS was not significantly different compared with sham controls (Fig. 2F). Following 5 weeks TAC, RWT was not different from sham controls but FS was significantly reduced compared with sham controls and BL/6J counterparts (all p < 0.05) (Fig. 2F). The reduction of FS was time-dependent (R^2^ = 0.661, p < 0.0001) (Fig. 2G). Following 5-weeks TAC, LW/BW ratio was also significantly increased in the BL6N mice (Fig. 2H) and all 5 mice exhibited heart failure (p = 0.009 and p = 0.021 compared with the 2 weeks TAC group and BL/6J counterparts, respectively) (Table 2). In contrast, following 2 weeks and 5 weeks TAC, BL/6J mice developed concentric hypertrophy as indicated by greatly increased HW/TL ratio (Fig. 2B) and RWT (Fig. 2C), and unchanged LV internal dimension (Fig. 2D, E). Cardiac function was preserved in the majority of BL/6J mice (Fig. 2F). These results clearly suggest that the duration of TAC (when less than 5 weeks) is an important factor affecting cardiac function in BL/6N mice but not in BL/6J mice.

**Table 2:** Heart failure rate in mice aged 8 weeks after 2-week and 5-week TAC.

TAC duration (week)	C57BL/6J	C57BL/6N	P value
2	20% (2/10)	25% (3/12)	1.000
5	25% (2/8)	100% (5/5)	0.021
P value	1.000	0.009	

Heart failure with reduced systolic function was defined as having dyspnea, body weight loss (approximately 10% decrease), increased wet lung weight and reduced fractional shortening on echocardiography compared with sham controls.

### 3.3 Variability of cardiac phenotypes in the C57BL/6J substrain

**Fig. 3:**
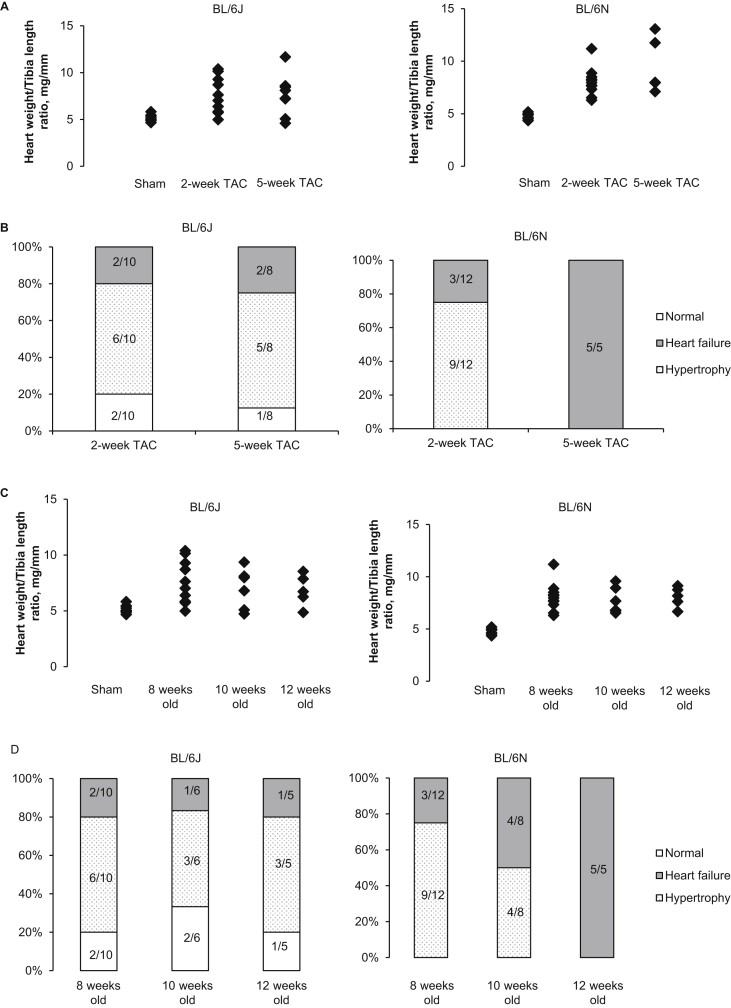
**Variability in hypertrophic response and cardiac phenotypes. (A)** Variability in heart weight to tibia length ratio in response to the duration of TAC. **(B)** Variability in cardiac phenotype in response to the duration of TAC. **(C)** Variability in heart weight to tibia length ratio in response to the age. **(D)** Variability in cardiac phenotype in response to the age. n = 5–12 per group. TAC, transverse aortic constriction.

Although the mean HW/TL ratio was not significantly different between the two substrains after 2-week and 5-week TAC (Fig. 2B), HW/TL ratio varied considerably in BL/6J mice (Fig. 3A). We used the greatest HW/TL ratio (5.83 mg/mm) in sham controls of both substrains as a cut-off value to determine which mice had not had a hypertrophic response to pressure overload. Our data showed that 20% (2/10) in the 2-week group and 25% (2/8) in the 5-week group of BL/6J TAC mice, whilst all BL/6N TAC mice exhibited hypertrophy (Fig. 3A). Both echocardiography and post-mortem examination verified that ligation of the aorta had been successful in all TAC mice. In general, BL/6J mice displayed variable cardiac phenotypes ranging from normal cardiac structure and function (without cardiac hypertrophy and failure), hypertrophy and heart failure independent of the duration of TAC. In contrast, BL/6N mice displayed hypertrophy and/or heart failure depending on the duration of TAC (Fig. 3B). Such variability in the hypertrophic response and cardiac phenotypes in BL/6J mice were also independent of age (Fig. 3C, D).

**Fig. 4:**
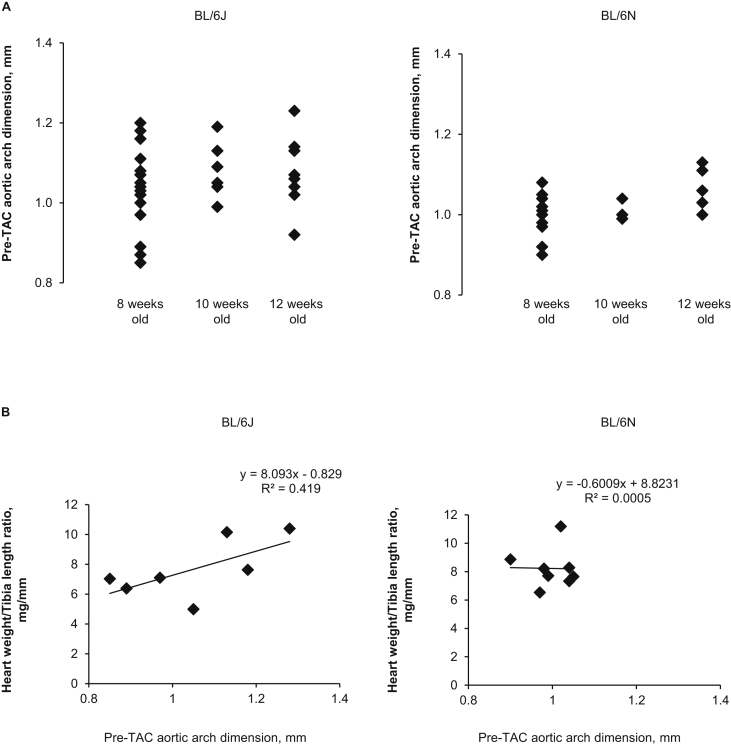
**Variability in pre-TAC aortic arch dimension and its relationship with the severity of hypertrophy. (A)** There is an age-independent variability in pre-TAC aortic arch dimension in C57BL/6J mice but not in C57BL/6NTac mice (n = 17–21 per group). **(B)** There is a positive correlation between the pre-TAC aortic arch dimension and HW/TL ratio in C57BL/6J mice (R^2^ = 0.419, p = 0.049) but not in C57BL/6NTac mice (n = 5–12 per group). TAC, transverse aortic constriction; HW/TL ratio, heart weight/tibia length ratio.

In order to determine the mechanisms underlying the variable phenotypes in BL/6J mice, we investigated the size of the aortic arch. In 8 week old mice, pre-TAC aortic arch dimension in the BL/6J substrain was highly variable, with a standard deviation of 0.107 compared with a standard deviation of 0.05 in the BL/6N substrain (p = 0.003, n = 17–21 per group). Such variability was consistently observed in all age groups (Fig. 4A). Furthermore, the pre-TAC aortic arch dimension positively correlated to the HW/TL ratio after 2 week TAC in the BL/6J substrain (R^2^ = 0.419, p = 0.049) but not in the BL/6N substrain (R^2^ = 0.001, p = NS) (Fig. 4B). These data suggest that BL/6J mice developed variable cardiac phenotypes depending on the degree of pressure overload, whilst the BL/6N substrain had less tolerance to pressure overload and developed cardiac hypertrophy and/or failure under any given level of pressure overload.

### 3.4 Strain-specific differences in cardiac remodelling after 2-week TAC

**Fig. 5:**
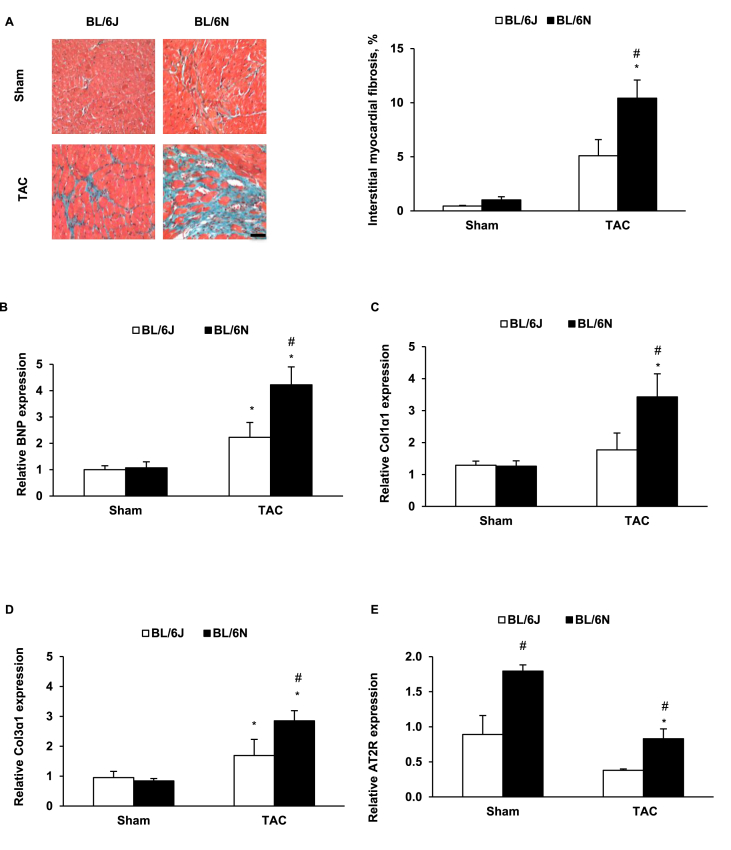
**Innate difference in cardiac remodelling after 2-week TAC. (A)** The percentage of the fibrosis area and representative images of Masson's trichrome staining in cardiac cross sections (magnification, ×20; bar = 100 μm) (n = 5–8 per group). **(B)** BNP mRNA expression (n = 5–8 per group). **(C)** Col 1α1 mRNA expression (n = 5–8 per group). **(D)** Col 3α1 mRNA expression (n = 5–8 per group). **(E)** AT2R mRNA expression (n ​= ​3–8 per group). *p ​< ​0.05 compared with the sham group of same strain. #p ​< ​0.05 compared with C57BL/6J mice at the same time point.

In order to assess cardiac remodelling before cardiac hypertrophy progresses from compensation to decompensation, we compared BL/6J and BL/6N TAC mice that had cardiac hypertrophy and preserved cardiac function. Although cardiac hypertrophy as assessed by HW/TL ratio (n = 6–11 per group) (Supplementary Figure 2A) and cardiomyocyte size (n = 5–8 per group) (Supplementary Figure 2B), and angiogenesis as assessed by capillary to cardiomyocyte ratio (n = 5 per group) (Supplementary Figure 2C) were not different between the two substrains (all p > 0.05), interstitial fibrosis was more profound in BL/6N mice than BL/6J counterparts (p = 0.044, n = 5–8 per group) (Fig. 5A). Conscious ECG showed a significant increase in QRS and QT intervals in both substrains following TAC (all p < 0.003) but the increase was greater in BL/6N TAC mice than in BL/6J counterparts (Table 3). No difference was found in haemodynamic assessment between the two substrains (Supplementary Table 1).

**Table 3:** Conscious electrocardiography before and after 2-week TAC in C57BL/6J and C57BL/6N mice.

	C57BL/6J			C57BL/6N			P (BL/6J vs. BL/6N TAC)
	Sham	2-week TAC	P	Sham	2-week TAC	P	
N number	11	6		7	9		
Heart rate, bpm	748 ± 12	759 ± 8	NS	735 ± 15	764 ± 12	NS	NS
RR interval, ms	80.3 ± 1.3	79.1 ± 0.8	NS	81.6 ± 1.7	78.7 ± 1.2	NS	NS
PR interval, ms	32.3 ± 0.7	33.0 ± 0.7	NS	31.5 ± 0.9	31.3 ± 1.2	NS	NS
P duration, ms	7.77 ± 0.60	8.95 ± 0.63	NS	6.45 ± 0.31	9.26 ± 0.33	0.000	NS
QRS interval, ms	10.4 ± 0.5	16.0 ± 0.5	0.000	12.2 ± 0.4	19.4 ± 1.3	0.000	0.009
QT interval, ms	22.9 ± 2.0	30.4 ± 0.7	0.006	24.8 ± 0.7	34.6 ± 1.8	0.000	0.045
QTc, ms	81.2 ± 7.2	108.0 ± 2.9	0.003	85.1 ± 2.2	124.3 ± 6.4	0.000	0.036
JT interval, ms	12.6 ± 1.5	14.4 ± 0.5	NS	12.7 ± 0.5	15.5 ± 0.9	0.038	NS

Measurements are represented as mean ± SE and compared with 2-tailed Student's *t*-test. TAC, transverse aortic constriction; NS, not significant.

In the TAC groups, we also found expected changes in gene expression of markers of pathological hypertrophy and fibrosis in cardiac tissue from both substrains. A comparison of mice of both substrains that had undergone TAC revealed that BL/6N mice had significantly greater expression of BNP (Fig. 5B), Col 1α1 (Fig. 5C) and Col 3α1 (Fig. 5D). We also investigated neurohormonal factors in these two C57BL/6 substrains. Notably, gene expression of AT2R in sham controls was significantly higher in BL/6N mice than that in BL/6J mice. After 2-weeks TAC, it was significantly down-regulated in BL/6N mice but the expression level was still higher than that in BL/6J mice (Fig. 5E). No difference was found in the gene expressions of angiotensin II type 1 receptor (Supplementary Figure 3A), or β1 and β2 adrenergic receptors (Supplementary Figure 3B, C). These results indicate that the influence of angiotensin II on cardiac remodelling may be different in the two C57BL/6 substrains.

## 4 Discussion

The present study is the first to systematically assess the impact of age and the duration of TAC on cardiac function in C57BL/6J and C57BL/6NTac substrains. It also further elucidated the nature of cardiac remodelling after 2-weeks exposure to TAC. We found that when both C57BL/6 substrains were subjected to the same degree of TAC, BL/6N mice developed eccentric hypertrophy with an age- and time course-dependent deterioration in cardiac function, whereas the BL/6J substrain developed variable cardiac phenotypes encompassing normal heart, concentric hypertrophy and heart failure. This variability was independent of age and the duration of TAC but likely dependent on the severity of pressure overload as evidenced by the correlation between HW/TL ratio and pre-TAC aortic arch dimension. Comparing mice with hypertrophy and preserved systolic function, BL/6N mice developed more fibrosis and higher expression of BNP and collagen type 1 and 3 genes than BL/6J mice after 2-week TAC. The expression of AT2R was also significantly higher in BL/6N mice compared with BL/6J mice at the basal level and after 2-week TAC.

Our knowledge about the impact of age on TAC-induced hypertrophy is mainly based on ageing (>12 months old) mice or rats (Li et al., 2003; Isoyama et al., 1992). Therefore, many investigators use mice aged between 6 and 16 weeks for TAC experiments. For the first time, we demonstrate that BL/6N mice are more sensitive to age-dependant cardiac dysfunction than BL/6J mice even in early adulthood. Systolic function significantly deteriorated in BL6N mice aged 10–12 weeks and heart failure was prominent in 12-week old mice when they had been subjected to 2-week TAC. Our findings corroborate those of Garcia-Menendez and co-workers who used 12 weeks old C57BL/6NTac mice to investigate the cardiac remodelling following TAC and found that LV systolic function was dramatically reduced after 2 weeks (Garcia-Menendez et al., 2013). The underlying mechanism for such an age-dependent effect on cardiac function in adult BL/6N mice has never been investigated. One of our studies has reported PDE4b structural variant in BL/6N mice (Simon et al., 2013). The function of PDE4b is to regulate Ca^2+^ cycling via PI3Kγ (Leroy et al., 2011) and PKA (Mika et al., 2014) signalling pathways and it plays critical roles in protecting against cardiac dysfunction and arrhythmias in mice. It is not clear whether this PDE4b variant is associated with age-dependent cardiac dysfunction under pressure overload-stress, in a similar way to another member of the PDE4 family, PDE4d (Lehnart et al., 2005). This requires further investigation.

In addition to the age effect, we also found that the impact of the duration of TAC on cardiac outcome is different between the two substrains. Eight weeks old BL/6N mice developed heart failure after 5-week TAC, an outcome that we also found in the C57BL/6NCrl substrain from Charles River (data not shown). In contrast, BL/6J mice have more resilience to TAC stimulation, with the majority of the mice maintaining normal cardiac function for a longer period (>5 weeks). Unlike BL/6N mice, BL/6J mice developed variable cardiac phenotypes independent of age and the duration of pressure overload. These findings are consistent with previous reports. Hermans et al. (Hermans et al., 2014) applied TAC to C57BL/6J mice aged over 10 weeks and found considerable diversity in the extent of cardiac remodelling after 2–10 weeks of TAC, including heart failure, hypertrophy and non-hypertrophy at each time point. Mohammed and colleagues (Mohammed et al., 2012) also reported that the severity of the changes in chamber geometry, systolic function, and hypertrophic and fibrotic remodelling were very variable in 8 week old C57BL/6J mice that had undergone 3 and 6 weeks TAC. However, the reasons underlying these discrepancies have never been reported. Here we found a considerable range in pre-TAC aortic arch dimension in the BL/6J substrain compared with the BL/6N substrain, which was independent of age but correlated with the severity of cardiac hypertrophy after 2-week TAC. This may explain why the deterioration of cardiac function in C57BL/6J mice can be observed in all age groups and irrespective of the duration of TAC (Garcia-Menendez et al., 2013; Sung et al., 2015; Zhang et al., 2013; Aksentijevic et al., 2010), whilst the cardiac phenotype in the BL/6N substrain was consistent from one mouse to the next. These findings are very important for future studies conducted in the BL/6 substrains. The BL/6J substrain appears to be an ideal model of sustained cardiac hypertrophy because cardiac function can be maintained for a significant period of time. However, the variability in cardiac phenotypes in BL/6J mice may mean large numbers of animals are required. Screening the aortic dimension by echocardiography before and after TAC may prove helpful to monitor and control such variabilities. The BL/6N substrain may be more suitable as a model of heart failure that can be induced within 5 weeks following TAC.

In addition to the age of the mice and the duration of TAC, the impact of genetic background is also a crucial factor during TAC-induced cardiac remodelling. Many investigators still treat C57BL/6 substrains as identical but a number of studies have shown that genetic differences between BL/6J and BL/6N substrains can affect phenotypes at baseline (Simon et al., 2013; Bothe et al., 2004) and after pathological stimulation (Garcia-Menendez et al., 2013; Cardin et al., 2014; Nickel et al., 2015). Garcia-Menendez et al. found marked fibrosis in 12 weeks old C57BL/6NTac mice after 8 weeks of TAC (Garcia-Menendez et al., 2013). Our results show that substantial fibrosis in BL/6N mice has already occurred by 2 weeks following TAC, which may accelerate cardiac dysfunction. The underlying mechanisms remain obscure. Nickel and co-workers showed more fibrosis and a greater incidence of heart failure in 10–12 weeks old C57BL/6NCrl mice compared with C57BL/6J mice after 6 weeks of TAC. They considered this was due to the pro-oxidative effect of the nicotinamide nucleotide transhydrogenase (Nnt) in the C57BL/6NCrl substrain because C57BL/6J mice lacking functional Nnt were protected from heart failure (Nickel et al., 2015). Apart from this, angiotensin II may also contribute to the difference in fibrosis between the two substrains. We found that gene expression of angiotensin II type 2 receptor is higher in BL/6N sham and TAC mice compared to their BL/6J counterparts. Studies have shown that angiotensin II type 2 receptors play important roles in the formation of fibrosis (Carey et al., 2000; Lemarie and Schiffrin, 2010). Using another model of cardiac hypertrophy induced by angiotensin II, Cardin et al. (Cardin et al., 2014) reported that chronic angiotensin II stimulation caused more interstitial fibrosis and affected a greater number of cardiac genes in the C57BL/6NCrl substrain compared with C57BL/6J, and these changes were independent of the mutation in Nnt. They did not measure the expression of AT2R; however, they did not find any difference in gene expression of AT1R between C57BL/6NCrl and C57BL/6J substrains. We therefore propose that the different effect of angiotensin II stimulation between the C57BL/6J and C57BL/6NCrl substrains may depend on the difference in AT2R. Further study is required to determine whether this is also the case in the C57BL/6NTac substrain.

It may be raised that a potential limitation to this study is that C57BL/6N mice have previously been reported to be more sensitive to the effect of anaesthesia under which they exhibit lower heart rates than C57BL/6J mice (Simon et al., 2013). However, we did not find any significant difference in heart rate between the two substrains under anaesthesia induced by 1% isoflurane. Therefore, we believe the differences between the two substrains are due to the genetic background. Another issue raised by TAC experiment is how to maintain the consistency of the aortic constriction, which can be affected by the tightness of constriction related to suture knots and fat tissue remaining around the aorta. Melleby and colleagues constrict the aorta using an o-ring with fixed inner diameters to achieve low intra- and inter-surgeon variation (Melleby et al., 2018). To guarantee the consistency of stenosis in our study, we cleared surrounding fat tissue and tightened the suture around the aorta and an overlying 27-gauge needle with two secure double overhand knots. Therefore, the influence from surgical technique alone was well controlled.

## 5 Conclusions

This is the first study to stratify the confounding effects of age and the duration of TAC on cardiac remodelling in order to identify optimal TAC models for two important C57BL/6 substrains. The present study provides important information for future studies using C57BL/6J and C57BL/6NTac mice to investigate TAC-induced hypertrophy and heart failure.

## CRediT author statement

**Min Zi:** Conceptualization, Methodology, Investigation, Writing – Original draft. **Nicholas Stafford:** Investigation, Writing – Review & editing. **Sukhpal Prehar:** Investigation. **Florence Baudoin:** Investigation. **Delvac Oceandy:** Writing – Review & editing. **Xin Wang:** Writing – Review & editing. **Thuy Bui:** Investigation. **Mohamed Shaheen:** Investigation. **Ludwig Neyses:** Funding acquisition. **Elizabeth J Cartwright:** Conceptualization, Funding acquisition, Writing – Review & editing.

## Declaration of Competing Interest

Authors have no conflicts of interest to disclose.
