# Supplementary material for: Cardiac hypertrophy or failure? - A systematic evaluation of the transverse aortic constriction model in C57BL/6NTac and C57BL/6J substrains
Source: Curr Res Physiol. 2019 Nov 2;1:1–10. doi: 10.1016/j.crphys.2019.10.001 (PMC7357793; doi:10.1016/j.crphys.2019.10.001)
Supplement: Multimedia component 1 [file mmc1.pptx]

## Slide 1
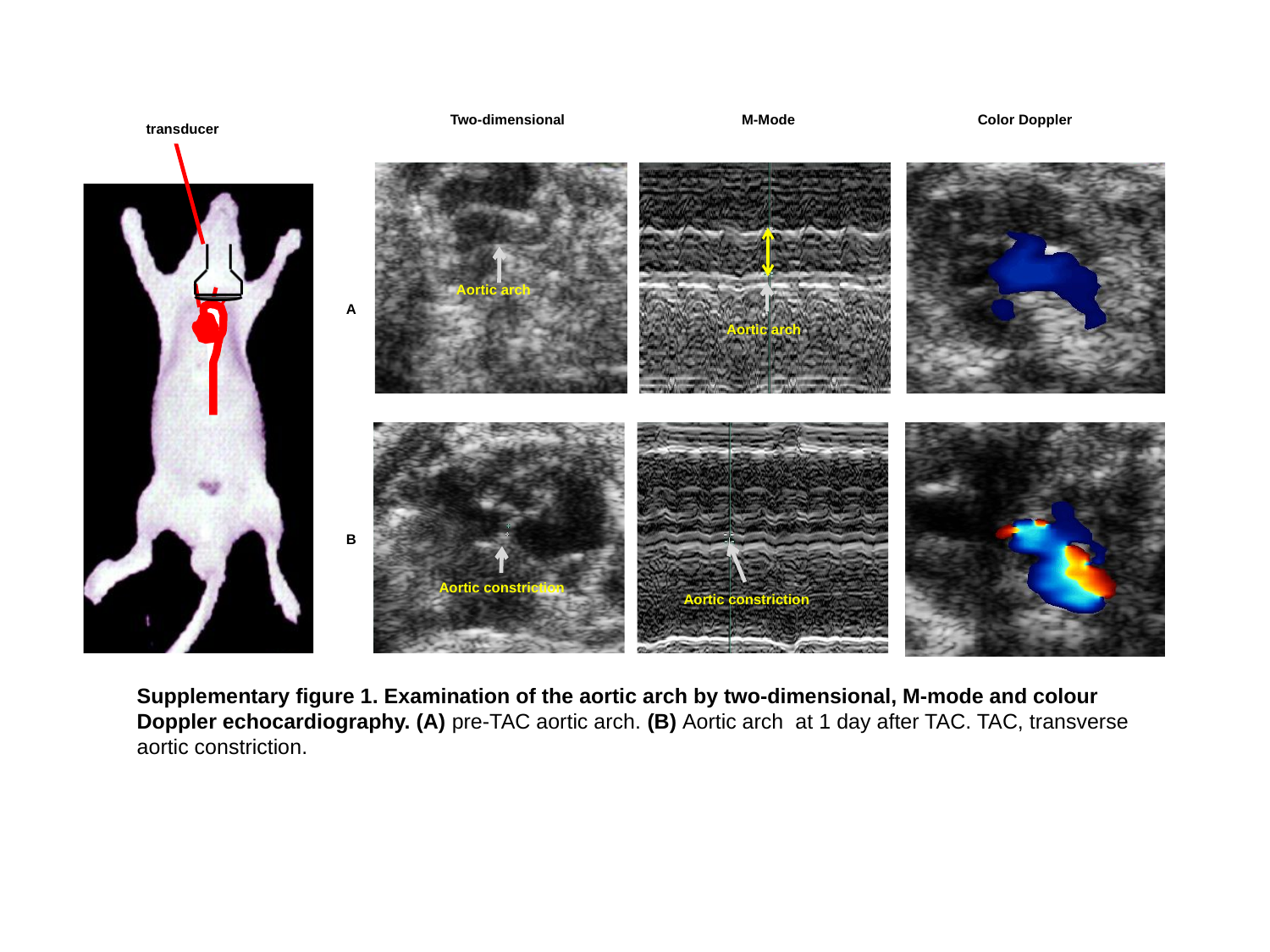

Two-dimensional
M-Mode
Color Doppler
transducer
Aortic arch
A
Aortic arch
B
Aortic constriction
Aortic constriction
Supplementary figure 1. Examination of the aortic arch by two-dimensional, M-mode and colour Doppler echocardiography. (A) pre-TAC aortic arch. (B) Aortic arch at 1 day after TAC. TAC, transverse aortic constriction.
