# Supplementary material for: Cardiac hypertrophy or failure? - A systematic evaluation of the transverse aortic constriction model in C57BL/6NTac and C57BL/6J substrains
Source: Curr Res Physiol. 2019 Nov 2;1:1–10. doi: 10.1016/j.crphys.2019.10.001 (PMC7357793; doi:10.1016/j.crphys.2019.10.001)
Supplement: Multimedia component 2 [file mmc2.pptx]

## Slide 1
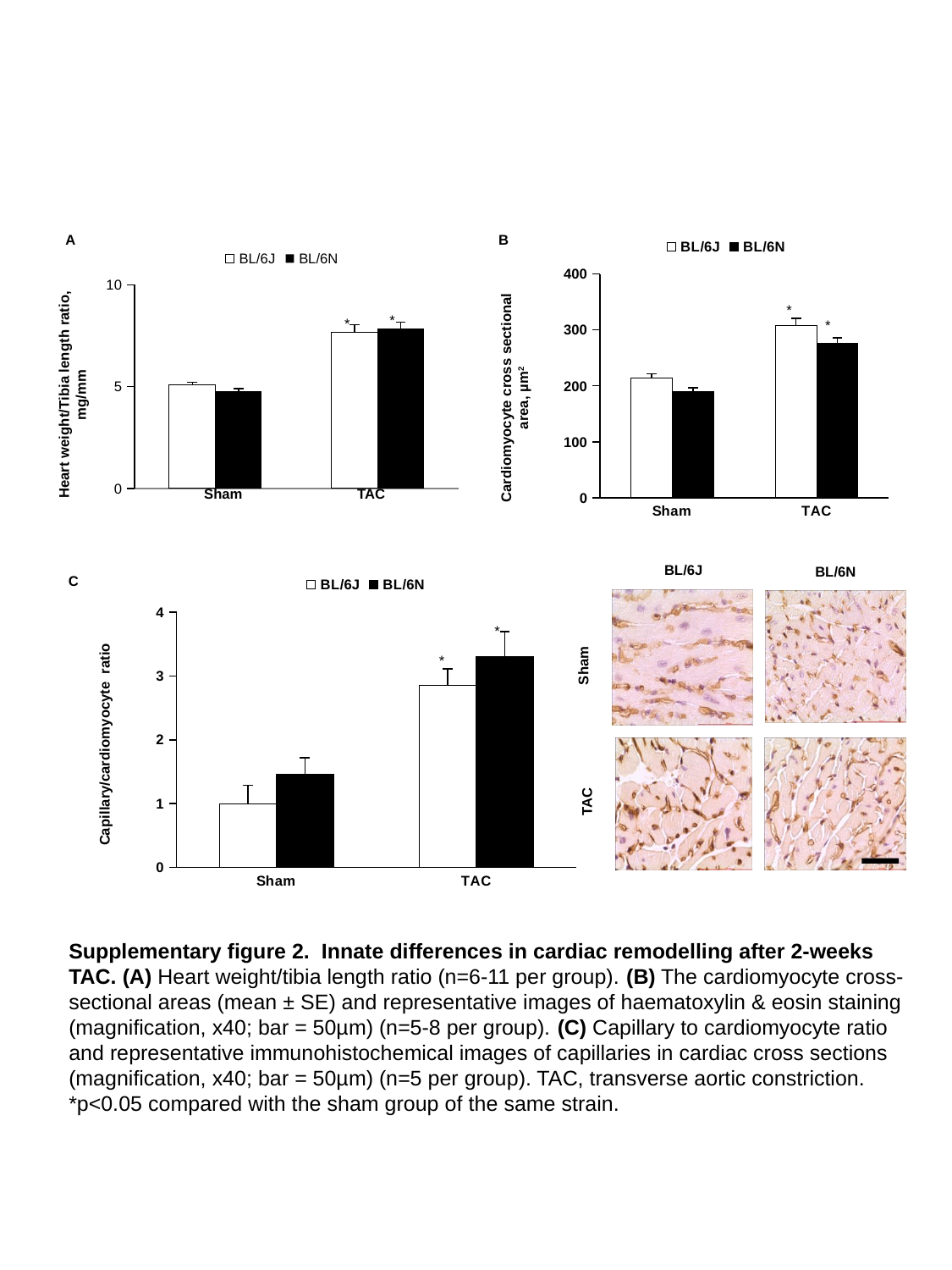

A
### Chart
| Category | BL/6J | BL/6N |
|---|---|---|
| Sham | 5.1 | 4.79 |
| 2-week TAC | 7.67 | 7.87 |Heart weight/Tibia length ratio, mg/mm
*
*
Sham
TAC
B
### Chart
| Category | BL/6J | BL/6N |
|---|---|---|
| Sham | 214.15 | 190.23 |
| TAC | 307.3399999999997 | 275.41 |Cardiomyocyte cross sectional area, µm2
*
*
BL/6J
BL/6N
### Chart
| Category | BL/6J | BL/6N |
|---|---|---|
| Sham | 1.0 | 1.46 |
| TAC | 2.8499999999999988 | 3.3 |C
Capillary/cardiomyocyte ratio
*
Sham
*
TAC
Supplementary figure 2. Innate differences in cardiac remodelling after 2-weeks TAC. (A) Heart weight/tibia length ratio (n=6-11 per group). (B) The cardiomyocyte cross-sectional areas (mean ± SE) and representative images of haematoxylin & eosin staining (magnification, x40; bar = 50µm) (n=5-8 per group). (C) Capillary to cardiomyocyte ratio and representative immunohistochemical images of capillaries in cardiac cross sections (magnification, x40; bar = 50µm) (n=5 per group). TAC, transverse aortic constriction. *p<0.05 compared with the sham group of the same strain.
