# Supplementary material for: Cardiac hypertrophy or failure? - A systematic evaluation of the transverse aortic constriction model in C57BL/6NTac and C57BL/6J substrains
Source: Curr Res Physiol. 2019 Nov 2;1:1–10. doi: 10.1016/j.crphys.2019.10.001 (PMC7357793; doi:10.1016/j.crphys.2019.10.001)
Supplement: Multimedia component 3 [file mmc3.pptx]

## Slide 1
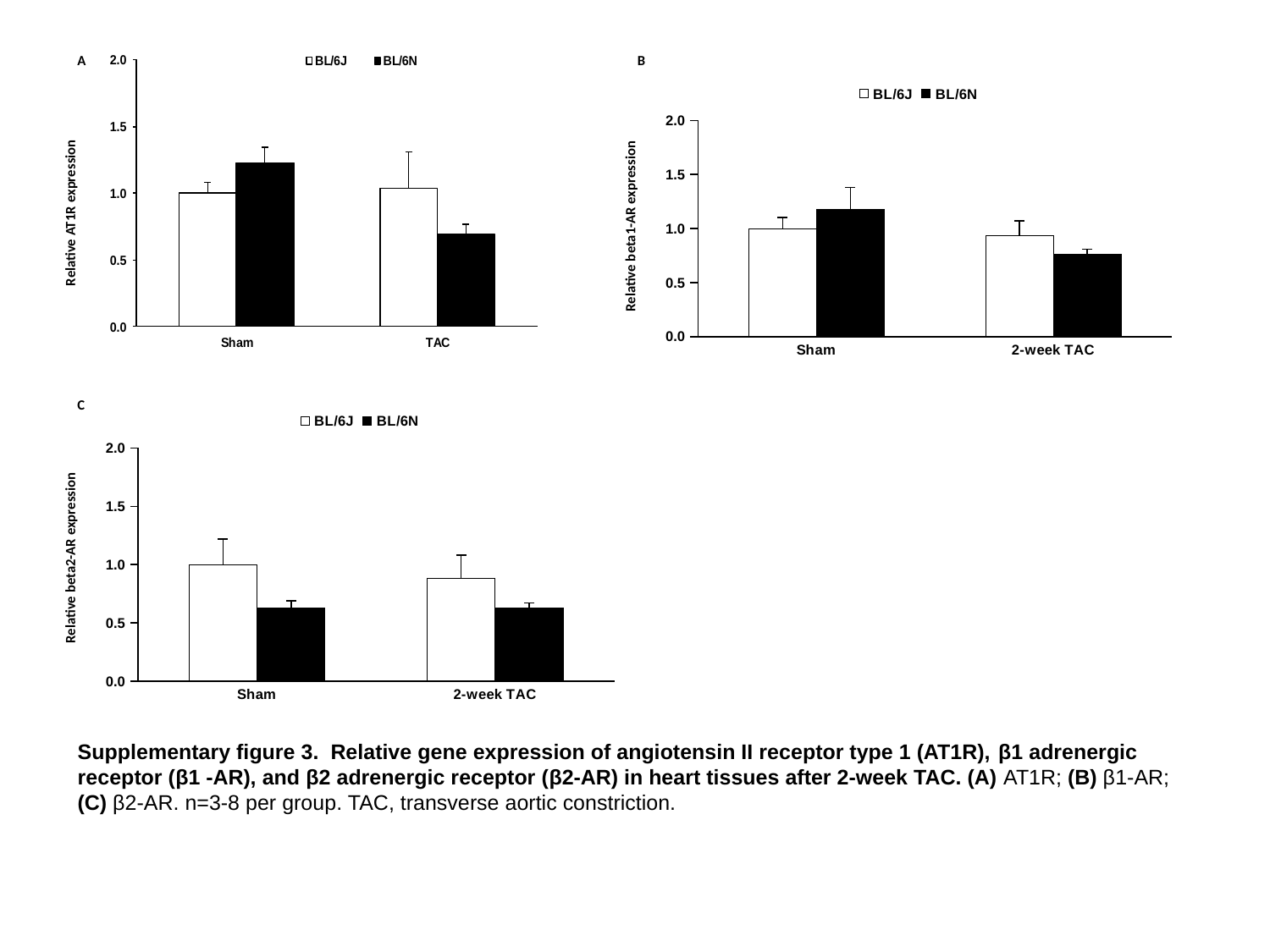

Relative AT1R expression
A
B
### Chart
| Category | BL/6J | BL/6N |
|---|---|---|
| Sham | 1.0 | 1.1800000000000008 |
| 2-week TAC | 0.93 | 0.7600000000000005 |Relative beta1-AR expression
Relative beta2-AR expression
C
### Chart
| Category | BL/6J | BL/6N |
|---|---|---|
| Sham | 0.9999999999999999 | 0.6300000000000009 |
| 2-week TAC | 0.8800000000000001 | 0.6300000000000009 |Supplementary figure 3. Relative gene expression of angiotensin II receptor type 1 (AT1R), β1 adrenergic receptor (β1 -AR), and β2 adrenergic receptor (β2-AR) in heart tissues after 2-week TAC. (A) AT1R; (B) β1-AR; (C) β2-AR. n=3-8 per group. TAC, transverse aortic constriction.
