# Supplementary material for: Cardiac hypertrophy or failure? - A systematic evaluation of the transverse aortic constriction model in C57BL/6NTac and C57BL/6J substrains
Source: Curr Res Physiol. 2019 Nov 2;1:1–10. doi: 10.1016/j.crphys.2019.10.001 (PMC7357793; doi:10.1016/j.crphys.2019.10.001)
Supplement: Multimedia component 4 [file mmc4.doc]

**Supplementary table 1. Haemodynamics after 2-week transverse aortic constriction**

|  | **BL/6J** | | | **BL/6N** | | | **P value** (BL/6J TAC vs. BL/6N TAC) |
| --- | --- | --- | --- | --- | --- | --- | --- |
|  | **Sham**  **(n=11)** | **TAC**  **(n=6)** | **P value** | **Sham**  **(n=7)** | **TAC**  **(n=9)** | **P value** |
| BW, g | 27.3±0.8 | 27.3±1.0 | NS | 26.0±0.6 | 27.2±0.5 | NS | NS |
| HR, bpm | 458±10 | 450±18 | NS | 449±13 | 459±9 | NS | NS |
| Right carotid SBP, mmHg | 88.6±3.0 | 150.6±9.6 | 0.000 | 76.5±1.9 | 148.5±9.0 | 0.000 | NS |
| dP/dt max, mmHg/s | 4826±383 | 5104±259 | NS | 3685±267 | 6852±2713 | NS | NS |
| dP/dt min, mmHg/s | 4275±540 | 4523±411 | NS | 3418±110 | 5237±834 | NS  (0.050) | NS |
| Tau, ms | 8.10±0.84 | 10.11±0.94 | NS | 8.80±0.32 | 6.67±1.20 | NS | NS (0.087) |
| ESPVR, mmHg/µl | 3.05±1.12 | 3.33±1.67 | NS | 3.74±1.53 | 5.35±1.14 | NS | NS |

Measurements are presented as mean ± SE and compared with 2-tailed Student's *t*-test. TAC, transverse aortic constriction; BW, body weight; HW, heart weight; SBP, systolic blood pressure; ESPVR, end systolic pressure volume relationship; NS, not significant.
